# Initial Validation and Psychometric Properties of the Croatian Version of the Pieper–Zulkowski Pressure Ulcer Knowledge Test

**DOI:** 10.3390/healthcare13131479

**Published:** 2025-06-20

**Authors:** Ana Žepina Puzić, Bojana Filej, Mirna Žulec, Vesna Bušac, Želimir Bertić, Anamarija Jurčev Savičević

**Affiliations:** 1 Department of Health Studies, Šibenik University of Applied Sciences, 22000 Šibenik, Croatia; vesna.busac@vus.hr; 2 Faculty of Health Sciences, University of Novo mesto, 8000 Novo mesto, Slovenia; bojana.filej@gmail.com; 3 Department of Nursing, Catholic University of Croatia, 10000 Zagreb, Croatia; mirna.zulec@gmail.com; 4 Department of Health Studies, University of Applied Sciences Ivanić Grad, 10300 Ivanić Grad, Croatia; bertic.z@gmail.com; 5 Faculty of Health Studies, University of Mostar, 88000 Mostar, Bosnia and Herzegovina; 6 Department of Nursing, Faculty of Health Studies, International University of Rijeka, 51000 Rijeka, Croatia; 7 Institute of Public Health of Bjelovar-Bilogora County, 43000 Bjelovar, Croatia; 8 University Department of Health Studies, Faculty of Health Sciences, University of Split, 21000 Split, Croatia; anamarija.jurcev.savicevic@nzjz-split.hr; 9 Teaching Public Health Institute of Split and Dalmatia County, 21000 Split, Croatia; 10 Medical Studies, School of Medicine University of Split, 21000 Split, Croatia

**Keywords:** validity, pressure injury, nursing, reliability, translation, cultural adaptation, nursing practice

## Abstract

**Background**: Understanding and preventing pressure ulcers is a key aspect of healthcare, with nurses playing a crucial role in their management and prevention through education and clinical practice to improve patient outcomes. **Objectives**: This quantitative, psychometric, cross-sectional study aimed to translate and culturally adapt the Pieper–Zulkowski Pressure Ulcer Knowledge Test and to evaluate the psychometric properties of the Croatian version of this measurement instrument. **Methods**: A study was conducted in a state hospital in Šibenik, Croatia, on a sample of 268 participants. **Results**: The content validity of the instrument, with an S-CVI of 0.981, indicates excellent validity, while its internal consistency is acceptable, as reflected in an overall KR-20 of 0.79. The maximum split-half reliability is 0.87 and Guttman Lambda 6 reaches 0.89, indicating excellent internal consistency. The overall test–retest reliability is excellent (ICC = 0.91), with the Prevention subscale showing the highest reliability (ICC = 0.88), followed by Staging (ICC = 0.86) and Wound Description (ICC = 0.84). Item-level difficulty and discrimination index were also calculated. **Conclusions**: The adapted, translated, and validated questionnaire represents a valuable tool for measuring knowledge about pressure ulcers among healthcare professionals, suitable for use in Croatian society. This instrument can be used to assess knowledge in clinical settings and evaluate the effectiveness of educational programs, providing valuable insights into the education and professional competence development of healthcare professionals.

## 1. Introduction

The treatment of pressure ulcers/injuries (PUs/PIs) remains a challenge worldwide [1] and is considered an indicator of quality of care [2]. Despite advancements in medicine, PUs/PIs still represent a ubiquitous health problem with multiple consequences on the health and treatment outcomes of patients, and they also constitute a large financial burden for health systems [3]. PUs/PIs developed in hospitals are financially debilitating [4] and are associated with prolonged hospitalization [5].

The clinical process of deciding how to treat PUs/PIs is delicate [6] and is dependent on the professional knowledge of nurses as well as the current available scientific evidence. Optimal decision making in this clinical process includes applying practice that is based on evidence with the use of health information technology [7]. The recommendations of current guidelines emphasize the need for regular assessment of the knowledge and attitudes of healthcare professionals to support clinical recommendations as well as to detect challenges [8], which can assist in implementing organizational and ergonomic strategies [9]. Formal and lifelong training programs for nurses on PU/PI prevention represent the key to effective prevention [10,11]. Interventions need to consist of evidence-based “portfolios” tailored to the needs of patients [12]. Nurses must be competent and well-educated in the prevention of PUs/PIs [13], ensuring the routine implementation of key strategies to improve mobility and reduce pressure [8].

The application of evidence-based healthcare (EBHC) [14] and evidence-based practice (EBP) in nursing is essential for effectively linking theoretical knowledge with practical patient care [15]. However, the results of previous studies indicate unsatisfactory levels of knowledge of nurses [16,17] and the need for regular knowledge checks for the purpose of defining educational needs and educational priorities [18]. The identified gap in the knowledge and competencies of healthcare workers should be assessed before the implementation of educational interventions and the adoption of the EBP process through the guidance of educational processes [19]. The use of high-quality, validated instruments to measure the knowledge, skills, and attitudes of healthcare professionals is essential for the effective implementation of EBP [20]. Although previous studies have mostly used self-assessment tools [21], tests that objectively measure knowledge are particularly important because they provide insight into actual competencies and allow for the monitoring of changes before and after educational interventions [22].

The Pieper–Zulkowski Test of Knowledge about pressure ulcers (PZ-PUKT) was developed by Barbara Pieper and Karen Zulkowski in 2014 and updated in 2021. This comprehensive tool assesses PU/PI knowledge in three domains: Prevention, Staging, and Wound Description [23].

This quantitative, psychometric, cross-sectional study was designed with the aim of translating and culturally adapting the PZ-PUKT and evaluating the psychometric properties of the Croatian version.

## 2. Materials and Methods

The aim of this cross-sectional psychometric study—performed in a state hospital in Šibenik, Croatia—was to conduct cultural adaptation, investigate internal consistency, and assess the psychometric properties of PZ-PUKT Version 2 among clinical nurses in Croatia. The STROBE guidelines for reporting observational studies were followed.

### 2.1. Instrument

In accordance with ethical guidelines, permission to use the instrument was obtained from the questionnaire designers via electronic correspondence. PZ-PUKT version 2 (2021) was used in this study. This instrument is an improved version of the original Pieper Pressure Ulcer Knowledge Test (PPUKT), one of the most commonly used knowledge assessment instruments [24] and recommended by the Clinical Practice Guidelines [25].

PZ-PUKT version 2 has been used in numerous studies (including cross-sectional, experimental, and interventional) and has been translated and validated in several languages, including Chinese, Polish, Portuguese, Persian, and Turkish [24,26,27,28,29], ensuring its applicability in different linguistic and cultural contexts. The instrument consists of 2 parts. The first part consists of 11 sociodemographic and educational items on the acquisition of knowledge about PUs/PIs. The second part of the questionnaire contains 72 items.

The second part of PZ-PUKT version 2 is divided into 3 subscales (Prevention, Wound Description, and Staging of PUs/PIs), containing 31, 20, and 21 items, respectively. There are a total of 35 correct answers in the questionnaire, with 37 incorrect ones. The possible answers are “True”, “False”, and “Do not know”. The test is scored by adding up the correct answers, and the range of possible correct answers is 0–72 points. Generally accepted scoring ranges for the original PZ-PUKT define less than 70% as unsatisfactory, 70% to 79.9% as satisfactory, 80% to 89.9% as good, and 90% and above as very good knowledge of pressure injury prevention [30].

### 2.2. Translation Procedure

The cultural adaptation of a psychometric instrument includes procedures that enable the adaptation of semantic, idiomatic, experiential, and conceptual components. The process of translation and adaptation of the PZ-PUKT questionnaire was conducted in accordance with the guidelines of Beaton and associates [31] in two phases.

The first phase involved forward translation, meaning the translation of the questionnaire from English to Croatian, which was performed by two translators—native Croatian speakers with excellent knowledge of English and an understanding of the goals and concepts of this study. Translator 1 is an associate professor (MD, PhD) with over 30 years of work experience. They provided opinions from a clinical perspective and their work resulted in a translation that ensured reliable equivalence from a measurement perspective. Translator 2 is an assistant professor (PhD) specializing in the field of Gerontology and Public Health with an M.A. of nursing, who played the role of a “naive translator” to ensure a different perspective on the original questionnaire than the first translator, offering a translation that reflects the language used by the Croatian population, and highlighting ambiguous meanings in the questionnaire. This phase resulted in two versions, T1 and T2. A translation synthesis was then conducted, where both translators and an observer synthesized the translation results. Working from the original questionnaire, together with the first and second versions of the questionnaire (T1 and T2, respectively), they created a joint translation (T12) in which the synthesis was carefully carried out through consensus. This was followed by back translation, during which two independent translators, who did not have access to the original questionnaire, translated the Croatian version back into English. This validation process ensured that the translated version reflects the same item content as the original version. This back-translation step is a type of validation used to spot conceptual errors in the translation. Back translations (BT1 and BT2) were performed by native speakers of the original language (English), who were not aware and not informed about the research concept to avoid bias and to discover unexpected meanings of the items in the translated questionnaire. The first translator was born in an English-speaking country, and the second one moved to an English-speaking country after formal education and has experience in translation. Both are fluent in English and Croatian.

After that, an expert committee was assembled with the aim of developing the pre-final version of the questionnaire, which was then tested. The expert group consisted of 4 members (an assistant professor, PhD; an associate prof., MD, PhD; an assistant professor, PhD; and a PhD candidate in Medical Sciences—M.A. of nursing) with knowledge of the topic and adopted methodology, as well as knowledge of Croatian and English, and 2 translators. The translation was analyzed in terms of semantic, idiomatic, experiential, and conceptual equivalence, and a consensus was reached. The report was compiled by the chairman of the expert committee and contained data on achieving equivalence between the original and the target (translated version).

In the second phase of this study, a pilot test was conducted on a sample of 30 nurses. The aim of the pilot was to assess general understanding and acceptance without the burden of testing construct validity. Participants completed a paper-based test, with each item accompanied by the following questions: “Is this expression clear to you?”, “Is there anything in this statement that you would change?”, and “Is this content appropriate for your daily work?” These questions were open- and closed-ended, and their purpose was to identify potential misunderstandings and terminological ambiguities. Participants were selected via purposive sampling from a group of nurses employed in different departments (surgical, internal medicine, and intensive care) of the same clinical institution. The inclusion criteria were active employees in clinical practice who worked with patients with PUs/PIs on a daily basis, and a minimum of one year of work experience. When recruiting the sample, diversity in terms of educational level (technician, bachelor, and master) and length of work experience was taken into account. The goal was to include participants with different levels of clinical experience in order to translate the instrument in a way that is understandable to a wide range of users. Participants had between 2 and 30 years of work experience. Different departments were represented, which ensured a diversity of professional contexts within the same institution. This was followed by a final revision of the instrument. 

**Figure 1:**
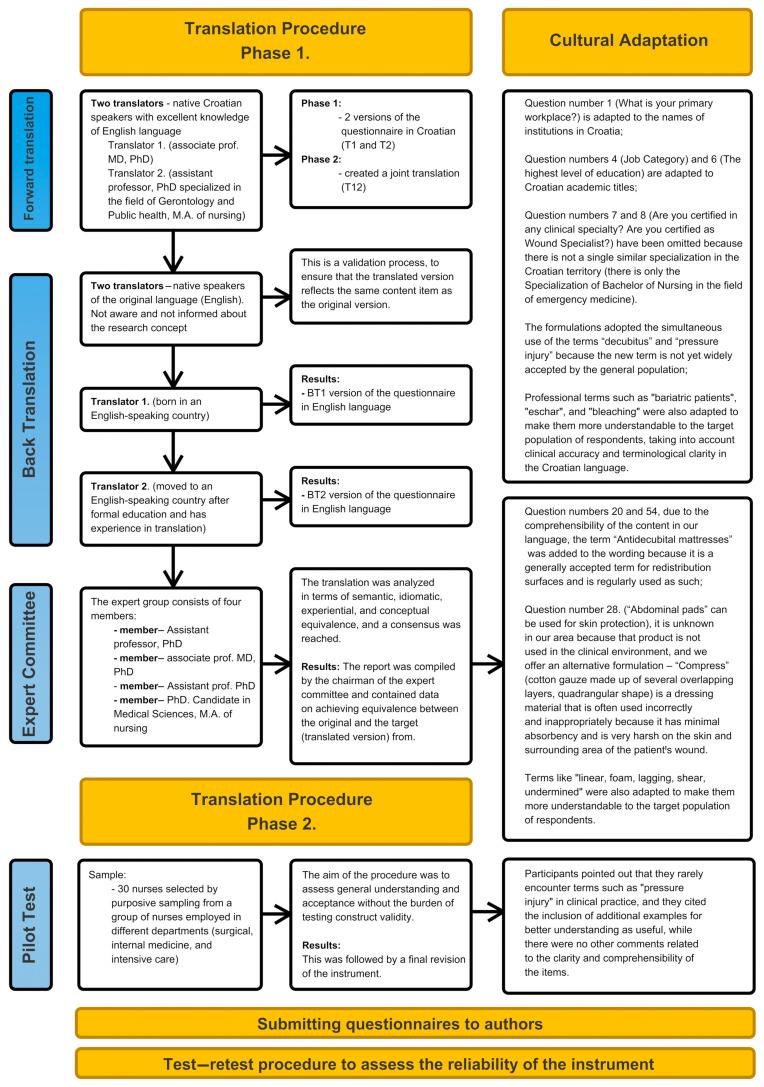
Translation procedure and cultural adaptation of the Pieper–Zulkowski Pressure Ulcer Knowledge Test.

After the pilot test, a final revision was carried out, and a final report with all versions was submitted to the authors of the questionnaire for review. The authors reviewed the reports and analyzed whether the translation and adaptation procedures of the instrument were carried out in a high-quality and satisfactory manner and whether a satisfactory translation was achieved. We obtained the authors’ consent for the procedure and changes carried out. This was followed by a test–retest procedure to assess the reliability of the instrument (Figure 1).

### 2.3. Assessment of Translation Equivalence

The method for assessing content validity developed by experts from The American Educational Research Association was used [32]. This study involved 12 experts who evaluated each item of the questionnaire using a Likert scale from 1 to 4 (where 1 indicates low relevance and 4 indicates high relevance). The group of experts consisted of six professors of nursing and six Masters of Nursing with proven expertise and experience in education and clinical practice who came from various health and educational institutions, including hospitals and higher education institutions. As educators and teachers, they were selected for their expertise and knowledge of the content relevant to the subject of the research. The selection criteria for the experts included at least 15 years of experience in clinical practice or education. Five of the experts had previous involvement in instrument validation, and the others had participated in the development of various educational materials. The experts provided feedback on the quality and clarity of each item and its importance for measuring the targeted construct. Their comments were considered and their suggestions were incorporated because they contributed to the simplification and clearer formulation of the items. After the revision, the final version of the questionnaire was again submitted to the experts for evaluation, whereby all modified items were rated with the highest rating. Thanks to their feedback, the wording of 3 items was adjusted to make them clearer, simpler, and more understandable for the target population.

The content validity of the PZ-PUKT was assessed using the Item-level Content Validity Index (I-CVI) and Scale Content Validity Index (S-CVI). Items with an I-CVI ≥ 0.78 were considered acceptable [33].

### 2.4. Methods of Assessing Validity and Reliability

To assess the reliability and psychometric properties of the instrument, the following tests were applied in the analysis: internal consistency assessed using the KR-20 coefficient, split-half reliability, test–retest reliability by subscales, and overall results, as well as difficulty index and discrimination index.

### 2.5. Data Collection

The phase of translation and cultural adaptation of the instrument was conducted from October 2023 to January 2024, with the pretest in February 2024. Data collection for the test and retest was conducted between March and May 2024 in printed form.

Each questionnaire form contained information about the purpose and objectives of this study, as well as the informed consent. The researcher supervised the completion of the questionnaire to ensure that the participants filled it out independently, without using any tools or external sources. The questionnaire of each participant was given a code, and their responses in the test and retest were linked through this code. On average, the questionnaire took 20 min to complete. No compensation was provided for participation in this study.

### 2.6. Sample

Determining the appropriateness of the sample size according to Bonett’s formula for sample size calculation [34] in testing and evaluating the α coefficient for a 72-item scale, the acceptable value for Cronbach’s α was 0.80. The confidence interval and test power were set at 95%. Based on these calculations, the minimum number of participants required for this study was 210.

A total of 300 questionnaires were distributed, and after excluding participants with incomplete questionnaires or a lack of response to retesting, 268 participants remained for analysis, representing a response rate of 89.33%. Participants from the pilot testing were not included in the total sample. The inclusion criteria were nurses employed in hospital departments with at least 1 year of experience, nurses of all education levels, and those who agreed to participate in the study. To reduce selection bias, we applied strict inclusion criteria, and we also used a random selection of participants in the groups to minimize bias. This study was conducted in one hospital (which covers a population of around 100,000 people) due to methodological and logistical reasons, as well as the availability of the target population and the operational feasibility of conducting the research. An additional reason for choosing this institution as a suitable location for conducting the research lies in the fact that the health education curriculum at the secondary and undergraduate levels in the Republic of Croatia is unique, which reduces the heterogeneity of respondents and increases the reliability of the collected data. Methodological reasons include the need for a controlled research environment, ensuring the consistent application of instruments, better operational feasibility, and the ability to monitor and supervise data collection. The selected hospital is a secondary-level health institution with 497 employed nurses, 20 hospital departments (Internal Department with General Internal Medicine, Pulmonology and Endocrinology with the Internal Medicine Intensive Care Unit, Hematology, Oncology, Allergology and Clinical Immunology; Cardiology with the Coronary Unit, Gastroenterology, Nephrology with hemodialysis; Department of Surgery with General Surgery, Plastic, Reconstructive and Aesthetic Surgery with Day Surgery, Abdominal Surgery, Pediatric and Vascular Surgery; and Units of Urology, Orthopedics and Traumatology, Otorhinolaryngology, Psychiatry, Neurology, Infectious Diseases, Dermatology and Venereology, Pediatrics and Anesthesia, Resuscitation and Intensive Care with Perioperative Nurses), and a developed subspecialty structure within large clinical units, thus reflecting the organization and clinical practice typical of the broader context of general, county, and clinical hospitals in the country. The sample was representative of healthcare professionals in different clinical settings and of different profiles, which contributes to the external validity and potential transferability of the findings to similar institutions in the same national context.

A stratification process was also carried out at the department level to ensure representativeness, with only those departments within the institution that treat high-risk patients and those with clinical conditions relevant to the research objectives being included in this study. The departments were selected based on their clinical activity, targeting departments with a high frequency of care for the target group of patients due to their clinical exposure to the changes that were the subject of the study. Across the relevant clinical departments, it was planned to ensure a minimum response rate of 50% from nurses in relation to the total number of employees in these departments, in an attempt to achieve a balanced representation of relevant clinical staff in the study, both in terms of their professional field and with regard to the age structure and length of service. The only organizational unit in which the participant response rate did not reach the planned level (it reached 30%) was the psychiatric unit. In this case, despite the efforts made, it was not possible to ensure the consistent participation of nurses in completing the instrument at both prescribed time intervals.

### 2.7. Data Analysis

Descriptive statistics were calculated to summarize sample demographics and professional characteristics. Continuous variables are presented as means with standard deviations (SDs) and medians with ranges, while categorical variables are expressed as frequencies and percentages. The content validity of the PZ-PUKT was assessed using the I-CVI and S-CVI. Twelve expert raters evaluated each item for relevance. Items with an I-CVI ≥0.78 were considered acceptable.

The internal consistency of the PZ-PUKT and its subscales was evaluated using the Kuder–Richardson formula 20 (KR-20). KR-20 values of 0.70 and above were considered acceptable. Additionally, split-half reliability was assessed using Guttman’s Lambda and the Spearman–Brown coefficient. Test–retest reliability was evaluated using the Intraclass Correlation Coefficient (ICC) with a two-way mixed-effects model to assess the consistency of test scores over time. The ICC values were reported with 95% confidence intervals, with values ≥0.75 indicating excellent reliability.

Item-level difficulty (DFI) and discrimination (DSI) indexes were calculated to assess the quality of individual test items. For the difficulty index (DFI), the proportion of correct responses for each item was categorized as easy (DFI > 0.70), moderate (0.30 ≤ DFI ≤ 0.70), and difficult (DFI > 0.30). For discrimination index (DSI): items were categorized as good (DSI ≥ 0.30), moderate (0.20 ≤ DSI < 0.30), marginal (0.10 ≤ DSI < 0.20), and poor (DSI < 0.10) based on their ability to differentiate between high- and low-performing participants. Statistical significance was set at *p* < 0.05, and confidence intervals were reported for key reliability measures.

All collected data were transferred to Microsoft Office Excel, where they were encoded for further processing, and statistical analyses were conducted using R software (version 4.3.1).

### 2.8. Research Ethics

The research was conducted in accordance with the ethical standards of the Declaration of Helsinki (version 2013). The participants were guaranteed anonymity and confidentiality, and to ensure the research complied with ethical principles, they were informed at the beginning of the study before giving their informed consent about the purpose and goals of the research, how their data would be used, and that their participation involved no risks. Participants could withdraw from the study at any time. This research was conducted using the paper version of the questionnaire. Permission to conduct the research was given by the Institution’s Ethics Committee prior to conducting the research (Class: 007-10/24-01/3, File Number: 2182-1-50-01-01-24-1).

## 3. Results

### 3.1. Basic Descriptive Demographic and Professional Characteristics of the Sample

Table 1 summarizes the demographic and professional characteristics of the sample (n = 268), which consisted primarily of female participants (97.4%). The average age was 38.4 years, with Master-level nurses being the oldest (42.8 years) and Bachelor-level nurses the youngest (35.1 years). Most participants worked in the department of surgery (28.7%), internal medicine (21.6%), or neurology (20.9%). The average professional experience was 15.5 years, with technicians and Master-level nurses having the longest experience.

### 3.2. Item-Level Content Validity Index

Table 2 presents the I-CVI for each item, categorized into three groups based on their I-CVI values. The assessment was conducted by 12 expert raters, with most items (59 out of 72) achieving perfect content validity (I-CVI = 1.00). Fourteen items had I-CVI values between 0.92 and 1.00, while one item (item 24) had a slightly lower I-CVI (0.83 ≤ I-CVI < 0.92). The overall S-CVI was 0.981.

### 3.3. Internal Consistency

Table 3 presents the internal consistency of the PZ-PUKT and its subscales using the KR-20. The overall scale reliability is 0.79. The Prevention subscale has a reliability of 0.68. Both the Wound Description and Staging subscales have a reliability of 0.57.

### 3.4. Split-Half Reliability

As shown in Table 4, the maximum split-half reliability is 0.87, Guttman Lambda 6 is 0.89, the average split-half reliability and Guttman Lambda 3 are both 0.80, Guttman Lambda 2 is 0.81, and the minimum split-half reliability is 0.60.

### 3.5. Test–Retest Reliability by Subscale and Overall Results

Table 5 presents the descriptive statistics and test–retest reliability with confidence intervals for the PZ-PUKT subscales and the overall scale. The overall test–retest reliability is ICC = 0.91. The Prevention subscale has a reliability of ICC = 0.88, followed by the Staging subscale at ICC = 0.86 and the Wound Description subscale at ICC = 0.84. The percentage of correct answers remains stable.

### 3.6. Categorized Difficulty Index

Table 6 categorizes the DFI of the test items into easy, moderate, and difficult based on the proportion of correct responses.

The categories with DFI > 0.70, between 0.30 and 0.70, and <0.30 comprise 33, 25, and 14 items, respectively.

### 3.7. Categorized Discrimination Index

Table 7 categorizes the DSI of items into four categories of good, moderate, marginal, and poor, based on their ability to differentiate between high- and low-performing respondents. The categories with a DSI ≥0.30, between 0.20 and 0.30, between 0.10 and 0.20, and <0.10 comprise 28, 12, 17, and 15 items, respectively.

### 3.8. Categorized Discrimination Index by Subscales

Table 8 presents the categorized discrimination indices (DSIs) for each subscale of the PZ-PUKT. Within the Prevention subscale, 12 items demonstrated good discrimination (DSI ≥ 0.30), while 8 items were moderate, 6 reasonable, and 5 poor. The Staging subscale also showed 12 items with good discrimination, 4 moderate, 2 reasonable, and 3 poor. Finally, in the Wound Description subscale, 9 items exhibited good discrimination and 8 moderate, with only 3 items in the reasonable range and none falling into the poor category.

## 4. Discussion

The methodological process adopted in the cultural adaptation of the Croatian version of PZ-PUKT, described in this study, was conducted in accordance with the scientific literature [31].

The content validity of the PZ-PUKT was assessed using the Item-level Content Validity Index (I-CVI) and Scale Content Validity Index (S-CVI). For this purpose, we engaged twelve experts from the field who assessed the appropriateness and relevance of each questionnaire formulation. The I-CVI was between 0.83 and 1.00. We calculated the S-CVI based on the average I-CVI calculated for each item of the instrument. The result of 0.981 indicated excellent content validity for the entire instrument [35]. The results from other validation studies indicate that the total S-CVI of the Turkish version was 0.960 (with an item range of 0.778 to 1), the Persian version had a total CVI index of 0.94, and all items of the Chinese version were corrected until they reached the maximum score [24,28,29].

The high S-CVI obtained in this study supports the clarity and objectivity of the statements of the instrument’s items in measuring the construct they are supposed to measure. Analyzing the results of previous psychometric studies in an international context, this instrument achieves the highest index values for the overall scale, proving that the experts assessed the items as being relevant for measuring the target construct.

The internal consistency of the instrument and its subscales was assessed using the Kuder–Richardson formula 20 (KR-20) for scales with dichotomous variables. The overall reliability of the instrument of 0.79 indicates acceptable internal consistency [36].

The Prevention subscale has a reliability of 0.68, and Wound Description and Staging each have a reliability of 0.57. The value of the Prevention subscale is at an acceptable level, while the remaining two subscales show moderate reliability and possible areas for improvement.

Comparing the results of other studies, it was found that for the Australian version, the KR-20 for the entire instrument was 0.86, while that of the Prevention subscale was 0.67, that of Wound Description was 0.76, and that of Staging was 0.65 [30]; other studies used Cronbach’s alpha to assess reliability. The Brazilian version had the lowest reliability coefficients of 0.42, 0.37, and 0.42 for the subscales, with a total Cronbach’s alpha of 0.82 [27]. For the Polish version as well, the reliability of the subscales was found to be below the acceptable level, at 0.50, 0.38, and 0.47, with a total Cronbach’s alpha of 0.72 [26]. The Philippine version was reported to have subscale reliability values of 0.56, 0.64, and 0.67, with an overall Cronbach’s alpha of 0.80 [37]. The best results were achieved by the Chinese version with subscale reliability values of 0.83, 0.84, and 0.82, with a total Cronbach’s alpha of 0.92 [24].

Analyzing the results of the internal consistency coefficient reported in different studies, it is evident that in general, the translated versions show acceptable reliability, while certain subscales could benefit from further refinement to improve internal consistency.

Internal consistency is calculated to determine the degree to which items within a test are correlated and consistently measure the same construct [38]. The maximum split-half reliability is 0.87 and Guttman Lambda 6 reaches 0.89, indicating excellent internal consistency. The average split-half reliability and Guttman Lambda 3 are both 0.80, suggesting good overall reliability. Guttman Lambda 2 provides a slightly more conservative estimate at 0.81, while the minimum split-half reliability, at 0.60, represents a lower bound for reliability. These results confirm the scale’s internal consistency and reliability, with some variation across different reliability estimates. Among the available translated and validated versions, only the Turkish version was examined with the Spearman–Brown split-half analysis, dividing the entire scale into two halves, and the results indicated satisfactory internal consistency with a test value of 0.889 [29].

Regarding the test–retest reliability of the PZ-PUKT, the overall reliability is excellent (ICC = 0.91), indicating strong consistency between test and retest scores. The Prevention subscale demonstrated the highest reliability among the subscales (ICC = 0.88), followed by Staging (ICC = 0.86) and Wound Description (ICC = 0.84). The percentage of correct answers remained stable between the test and retest for all subscales, with minor variations. These results confirm the high reproducibility of the PZ-PUKT across time, with narrow confidence intervals for all reliability estimates. Compared to our study, the Turkish study reported similar but slightly weaker reliability results, with high correlations ranging from 0.746 to 0.871 [29].

The analysis of the instrument items was carried out using the difficulty index and the discrimination index. The aim of this process is to clarify reliability and potential dilemmas, and it represents a key step for assessing the validity of individual test items [39]. The difficulty index (DFI) is calculated based on the percentage of correct answers in relation to the total number of responses, indicating the ease or difficulty of individual items [40]. It represents the percentage of participants who answered a specific test item correctly. An item with a difficulty index of less than 0.30 is considered difficult, while an item with a value higher than 0.70 is considered easy, and the desired range falls between 0.30 and 0.70 [41].

The difficulty index (DFI) of test items can be categorized into easy, moderate, and difficult based on the proportion of correct responses. In this study, easy items (DFI > 0.70) comprise the largest group, with 33 items, indicating they were answered correctly by most respondents. Moderate items (0.30 ≤ DFI ≤ 0.70) account for 25 items, representing an optimal range of difficulty. Difficult items (DFI < 0.30) include 14 items, suggesting that these were the most challenging for participants. The distribution of difficulty is reasonably balanced, with a number of items across different difficulty levels.

The discrimination index of an item shows how well the item distinguishes between high- and low-scoring participants [42]. To determine the discrimination index of the items, the participants were divided into two groups: the top 27% of performers and the bottom 27% of performers [43].

The categorization of the discrimination index (DSI) of items into good, moderate, reasonable, and poor categories is based on their ability to differentiate between high- and low-performing respondents. Analyzing the results of the discrimination index, it can be concluded that 40 items exhibited effective and acceptable discrimination in distinguishing performance levels, with DSI values ranging from 0.20 to ≥0.30. The group of items with reasonable DSI values (0.10 ≤ DSI < 0.20) includes 17 items, suggesting that these items may require a smaller review or revision. Finally, the group of poor items (DSI < 0.10), comprising 15 items, are prime candidates for review.

Additional analysis of the DSI of the PZ-PUKT by subscale provided a more detailed insight into the psychometric properties of the individual items. The results show an improved ability of this analysis to identify items with insufficient discriminative properties, with the total number of items with poor DSI values being significantly lower, especially when compared to the analysis at the level of the overall scale.

For the Prevention subscale, 26 items showed discrimination in the range of good to reasonable, while 5 items had poor discrimination. The items with poorer DSI results were “Seating should be for short periods in an appropriate chair/wheelchair with a pressure redistribution cushion for persons at risk for pressure ulcers/injuries”, “Reposition individuals with or at risk of pressure ulcer/injury on an individualized schedule regardless of mobility level unless contraindicated”, “It is the nurse’s responsibility to be sure a specialty bed is working properly and document its use”, “Massage of bony prominences is essential for quality skin care”, and “Persons, who are immobile and can be taught, should shift their weight every 30 min while sitting in a chair”. The Staging subscale showed a similar pattern, with 18 items ranging from good to reasonable discrimination and 3 items of poor discrimination. The items that had poorer DSI results were “Pressure ulcers/injuries progress in a linear fashion from Stage 1 to 2 to 3 to 4”, “Non-blanchable erythema anywhere in the body is a stage 1 pressure ulcer/injury”, and “If necrotic tissue is present and if bone can be seen or palpated, the ulcer is a Stage 4”. The most favorable results were obtained for the Wound Description subscale, where all 20 items were in the range of good to reasonable discrimination, and no item was categorized as poor.

The poorer DSI results of some items might have been due to a combination of conceptual complexity, different institutional procedures and distributions of responsibilities (e.g., differences in work organization and division of responsibilities), and inconsistency in clinical practice with updated clinical guidelines. Instead of reflecting a flaw in the validity of the items, they might have arisen due to a lack of knowledge among nurses and weaknesses in the education of the surveyed population regarding PUs/PIs, which is consistent with the findings of a similar study in Iran.

### 4.1. Strengths of the Study

This cross-sectional study provided the first validated questionnaire for assessing healthcare professionals’ knowledge of pressure ulcers in Croatia, covering three factors: Prevention, Wound Description, and Staging. Nurses are in a unique position to provide healthcare, as they are authorized through their competencies to implement preventive measures and to classify and treat pressure ulcers/injuries. This study yielded positive findings regarding the PZ-PUKT instrument as a standardized tool for assessing nurses’ knowledge, as well as the knowledge of other healthcare professions, contributing to the delivery of effective and purposeful care. Furthermore, certain subscales, such as the Prevention subscale, may be utilized independently from the full instrument to evaluate the knowledge level of non-healthcare personnel, such as caregivers.

### 4.2. Limitations of the Study

A limitation of this study is the presence of items with low discrimination power, the need for potential revisions of certain test items, and challenges related to item difficulty. Despite a generally balanced distribution of difficulty levels, these factors highlight areas for improvement. Another limitation of this study is the fact that it was conducted in only one public hospital, and since it is a cross-sectional study, the results may not be generalizable, as the level of knowledge changes through formal and lifelong learning.

## 5. Conclusions

The aim of this study was to conduct the adaptation, translation, and validation of a standardized and improved version of the Pieper–Zulkowski questionnaire, as well as to analyze its internal consistency, in order to create a valuable tool for measuring knowledge about pressure ulcers that is suitable for use in Croatian society. Cultural and contextual adjustments were made throughout the translation process, including the harmonization of professional terminology and expressions specific to the Croatian healthcare system, to make the instrument as relevant and understandable as possible to healthcare professionals in the local context.

Analytical procedures such as content validity, internal consistency, test–retest reliability, item-level difficulty, and discrimination indexes, conducted to assess the quality and reliability of the instrument, indicate satisfactory results.

The adapted, translated, and validated instrument can be used to assess knowledge in healthcare settings as well as in social and educational institutions to evaluate the effectiveness of educational programs, providing valuable insights into healthcare professionals’ competencies. It allows for an objective assessment of the level of knowledge of healthcare professionals on the prevention, classification, and description of pressure ulcers, which provides a basis for planning targeted educational interventions, assessing their effectiveness, and identifying specific areas that require additional professional development. In the research context, this instrument provides a validated and reliable measure for assessing the impact of educational program interventions and allows for a comparison of data across healthcare institutions and over time. The data obtained from this instrument can be used to develop formal and lifelong educational programs, targeted guidelines, and training on the topic, thereby further enhancing the quality of care provided by healthcare workers and their professional development.

## Figures and Tables

**Table 1:** Basic descriptive demographic and professional characteristics of the sample.

	Technician (n = 113)	Bachelor (n = 102)	Master (n = 53)	Overall (n = 268)
Gender				
Male	5 (4.4%)	2 (2.0%)	0 (0%)	7 (2.6%)
Female	108 (95.6%)	100 (98.0%)	53 (100%)	261 (97.4%)
Age				
Mean (SD)	39.2 (13.0)	35.1 (8.60)	42.8 (8.86)	38.4 (11.0)
Median [Min, Max]	39.0 [20.0, 64.0]	34.5 [22.0, 63.0]	43.0 [25.0, 62.0]	38.0 [20.0, 64.0]
Department				
Surgery	49 (43.4%)	21 (20.6%)	7 (13.2%)	77 (28.7%)
Internal	16 (14.2%)	28 (27.5%)	14 (26.4%)	58 (21.6%)
Neurology	18 (15.9%)	22 (21.6%)	16 (30.2%)	56 (20.9%)
ICU	6 (5.3%)	13 (12.7%)	3 (5.7%)	22 (8.2%)
Anesthesia	1 (0.9%)	4 (3.9%)	2 (3.8%)	7 (2.6%)
Psychiatry	2 (1.8%)	0 (0%)	1 (1.9%)	3 (1.1%)
Pediatrics	16 (14.2%)	10 (9.8%)	9 (17.0%)	35 (13.1%)
OBGYN	2 (1.8%)	1 (1.0%)	0 (0%)	3 (1.1%)
Operating room nurses	3 (2.7%)	3 (2.9%)	1 (1.9%)	7 (2.6%)
Position				
Technician	113 (100%)	12 (11.8%)	4 (7.5%)	129 (48,1%)
Bachelor	0 (0%)	87 (85.3%)	17 (32.1%)	104 (38,8%)
Master	0 (0%)	3 (2.9%)	32 (60.4%)	35 (13.1%)
Experience				
Mean (SD)	17.3 (12.6)	12.3 (8.52)	17.7 (10.1)	15.5 (11.0)
Median [Min, Max]	17.0 [1.00, 43.0]	10.0 [1.00, 42.0]	16.0 [3.00, 40.0]	15.0 [1.00, 43.0]

**Table 2:** Item-level Content Validity Index.

I-CVI	Items	Item Count
I-CVI = 1.00	2, 3, 4, 5, 6, 7, 8, 9, 10, 12, 13, 14, 15, 16, 17, 18, 19, 22, 23, 26, 27, 29, 30, 31, 34, 35, 37, 38, 39, 40, 42, 43, 44, 45, 46, 47, 48, 50, 51, 52, 53, 54, 55, 57, 58, 59, 60, 61, 62, 63, 64, 65, 66, 67, 68, 70, 71	57
0.92 ≤ I-CVI < 1.00	1, 11, 20, 21, 25, 28, 32, 33, 36, 41, 49, 56, 69, 72	14
0.83 ≤ I-CVI < 0.92	24	1

**Table 3:** Internal consistency (reliability) of subscales and overall scale.

Scale	KR-20
PZ-PUKT Prevention	0.68
PZ-PUKT Wound Description	0.57
PZ-PUKT Staging	0.57
PZ-PUKT Overall	0.79

**Table 4:** Split-half reliability results.

Statistic	Value
Maximum Split-Half Reliability	0.87
Guttman Lambda 6	0.89
Average Split-Half Reliability	0.80
Guttman Lambda 3 (Cronbach’s Alpha)	0.80
Guttman Lambda 2	0.81
Minimum Split-Half Reliability (Beta)	0.60

**Table 5:** Test–retest reliability by subscale and overall results.

	% of Correct; Mean (SD)				
Factor	Test	Retest	ICC	LCI	UCI
PZ-PUKT Prevention	65.7 (12.1)	65.1 (11.6)	0.88	0.85	0.90
PZ-PUKT Wound Description	54.7 (14.3)	55.6 (13.5)	0.84	0.79	0.87
PZ-PUKT Staging	56.1 (13.6)	56.3 (12.9)	0.86	0.83	0.89
PZ-PUKT Overall	59.8 (10.8)	59.9 (9.99)	0.91	0.88	0.93

Legend: ICC = interclass correlation; LCI = 95% lower confidence interval; UCI = 95% upper confidence interval.

**Table 6:** Categorized difficulty index.

DFI Category	Items	Count
Easy (DFI > 0.70)	1, 2, 5, 8, 9, 11, 15, 16, 17, 18, 19, 20, 22, 23, 24, 27, 29, 31, 32, 33, 45, 46, 47, 48, 50, 52, 56, 57, 58, 60, 62, 64, 71	33
Moderate (0.30 ≤ DFI ≤ 0.70)	3, 6, 7, 12, 13, 14, 25, 28, 30, 35, 36, 37, 38, 40, 43, 49, 53, 55, 59, 61, 63, 66, 68, 69, 70	25
Difficult (DFI < 0.30)	4, 10, 21, 26, 34, 39, 41, 42, 44, 51, 54, 65, 67, 72	14

**Table 7:** Categorized discrimination index for the overall instrument.

DSI Category	Items	Count
Good (DSI ≥ 0.30)	2, 6, 18, 19, 20, 21, 24, 25, 26, 28, 29, 31, 33, 35, 40, 43, 45,46, 47, 50, 53, 54, 55, 59, 61, 67, 68, 70	28
Moderately (0.20 ≤ DSI < 0.30)	7, 14, 17, 30, 36, 37, 56, 58, 60, 64, 65, 69	12
Reasonable (0.10 ≤ DSI < 0.20)	1, 3, 4, 5, 11, 13, 16, 23, 32, 34, 38, 42, 48, 57, 62, 66, 72	17
Poor (DSI < 0.10)	8, 9, 10, 12, 15, 22, 27, 39, 41, 44, 49, 51, 52, 63, 71	15

**Table 8:** Categorized discrimination index by subscale.

Subscale	DSI Category	Items	Count
PRE	Good (DSI ≥ 0.30)	19, 20, 24, 26, 29, 31, 45, 46, 47, 54, 55, 59	12
PRE	Moderate (0.20 ≤ DSI < 0.30)	13, 16, 17, 36, 56, 57, 58, 62	8
PRE	Reasonable (0.10 ≤ DSI < 0.20)	4, 9, 15, 23, 34, 66	6
PRE	Poor (DSI < 0.10)	5, 8, 27, 44, 51	5
STG	Good (DSI ≥ 0.30)	6, 14, 21, 25, 33, 35, 40, 43, 49, 50, 53, 69	12
STG	Moderate (0.20 ≤ DSI < 0.30)	37, 63, 64, 72	4
STG	Reasonable (0.10 ≤ DSI < 0.20)	32, 52	2
STG	Poor (DSI < 0.10)	10, 12, 22	3
WD	Good (DSI ≥ 0.30)	3, 7, 28, 30, 38, 61, 65, 68, 70	9
WD	Moderate (0.20 ≤ DSI < 0.30)	1, 2, 11, 18, 42, 60, 67, 71	8
WD	Reasonable (0.10 ≤ DSI < 0.20)	39, 41, 48	3
WD	Poor (DSI < 0.10)		0

Legend: PRE = Prevention subscale; STG = Staging subscale; WD = Wound Description subscale.

## Data Availability

The data presented in this study are available from the corresponding author upon request.

## Abbreviations

The following abbreviations are used in this manuscript:

PZ-PUNKT	Pieper–Zulkowski pressure ulcer knowledge test
PU/PI	Pressure ulcer/pressure injury
SD	Standard deviations
I-CVI	Item-level content validity index
S-CVI	Scale content validity index
KR-20	Kuder–Richardson formula 20
ICC	Intraclass correlation coefficient
DFI	Difficulty index
DSI	Discrimination index
EBHC	Evidence-based healthcare
EBP	Evidence-based practice
